# San Antonio’s Invitation to the A. M. A.

**Published:** 1902-06

**Authors:** 


### SAN ANTONIO'S INVITATION TO THE A. M. A.

The West Texas Medical Association and the San Antonio Business Men's Club are together making a determined effort to secure the 1903 meeting of the American Medical Association. The officers and committees representing these organizations are receiving encouragement and generous support from many prominent citizens, including Mayor Hicks, and from the local press. Governor Sayers has already endorsed the invitation which is to be presented to the House of Delegates at the Saratoga convention (meeting), and has promised to write a special note of invitation to be read before that body.

The Texas State Medical Association, while in session at Dallas, adopted a resolution instructing its delegates, Drs. H. A. West and J. T. Wilson, to use their influence in favor of San Antonio.

It seems, therefore, that if the National Association can be induced to come south for its next meeting, San Antonio's chances for being selected as the place for that meeting are exceedingly good.

San Antonio's ability to entertain the Convention and to make a good impression for Texas can not be questioned. The largest and most beautiful city in the State, it offers ample hotel accommodations, a number of well constructed and commodious halls, one of which will accommodate 5,000, and in addition, a better climate and more points of historic interest than any other place of its size in the South.

The city has been fortunate during the past few years in having an administration both honest and progressive, with one result, among others, that the streets, parks and plazas have been wonderfully improved and made clean and attractive.

The railroads are willing to give the usual reduction in rates, and to grant an exceptionally low excursion rate to all points in Mexico.

The many advantages that San Antonio offers as a meeting place for the National Association, taken all together, should far outweigh the single possible disadvantage of its being situated in the extreme southwestern part of the map. W. B. R.

